# Lived experiences of people living with HIV: a descriptive qualitative analysis of their perceptions of themselves, their social spheres, healthcare professionals and the challenges they face daily

**DOI:** 10.1186/s12889-021-10881-y

**Published:** 2021-05-12

**Authors:** Gamze Senyurek, Mustafa Volkan Kavas, Yesim Isil Ulman

**Affiliations:** 1 Department of Medical Humanities, Amsterdam UMC, De Boelelaan 1089a |, 1081 HV Amsterdam, Netherlands; 2 Department of History of Medicine and Ethics, Faculty of Medicine, Ankara University, Morfoloji Binasi, Tip Tarihi ve Etik AD. 06230, Altindag, Ankara, Turkey; 3 Department of History of Medicine and Ethics, School of Medicine, Acibadem Mehmet Ali Aydinlar University, Kayisdagi Cad. No:32, Atasehir, Istanbul, Turkey

**Keywords:** HIV, Stigmatization, Confidentiality, Discrimination, Lived experience, Access to healthcare, Turkey

## Abstract

**Background:**

Human immunodeficiency virus (HIV) infection rates have been gradually increasing in Istanbul, Turkey. Many people living with HIV (PLWH) here encounter difficulties, for example, in adapting to the chronic disease and obtaining continuous access to healthcare services. In this study, we aimed to explore the challenges PLWH face in their daily lives and understand their perceptions of themselves, healthcare professionals and services, and their social spheres via their expressed lived experiences in the healthcare setting.

**Method:**

Individual semi-structured in-depth interviews were conducted face-to-face with 20 PLWH in Istanbul. All the interviews were voice-recorded and transcribed verbatim except one, upon participant request, for which the interviewer took notes. These logs and the interviewer’s notes were analyzed thematically using the inductive content analysis method.

**Results:**

The themes concerned experiences in three distinct contexts: 1) Interactions with healthcare providers; 2) Participants’ responses to their HIV diagnosis; and 3) Interactions with their social networks. Firstly, the results highlighted that the participants perceived that healthcare professionals did not inform them about the diagnosis properly, failed to protect patients’ confidentiality and exhibited discriminative behaviors towards them. Secondly, after the diagnosis the participants had difficulty in coping with their unsettled emotional state. While many ceased sexual activities and isolated themselves, some sought support. Lastly, living with HIV affected their relationships with their families and friends either positively or negatively. Moreover, they had to face the difficulties concerning spouse/partner notification issues about which many needed professional support.

**Conclusion:**

Healthcare professionals’ discriminative or inappropriate attitudes and customs in healthcare institutions are perceived to impair PLWH’s utilization of healthcare services. Structural factors such as social pressure, societal ignorance about HIV, limited access to HIV prevention, and regulatory barriers might contribute to these challenges. The results suggest that it is necessary to raise healthcare professionals’ and society’s awareness about HIV and develop national policies to establish a well-functioning referral system and appropriate spouse/partner notification services.

## Background

Turkey is one of the countries in Eastern Europe and Central Asia wherein cases of human immunodeficiency virus (HIV) significantly increased between 2005 and 2014 [1]. While the prevalence of HIV in the country was low, recent data published by the Ministry of Health (MoH) show that the rate is surging [2]. According to the MoH records, the incidence of HIV infection was first recorded in 1985, when the total number of people living with HIV (PLWH) was just three. Twenty years later, the official number of PLWH in Turkey had increased to 168. Over the last few decades, exponential growth has been seen, with the total number of PLWH at 26,164 in 2020 [3].

The first relevant legislative regulations were implemented in 1985 when the first case was recorded. Since then, it is mandatory to notify HIV cases to the MoH. HIV testing was made a mandatory prerequisite for health-related applications, such as transfusion of blood and blood products in 1986 and for tissue/organ donation and registration of sex workers in 1987. In the following years, HIV testing was also included in mandatory pre-marital tests, tests performed during pregnancy, and the screening procedures before surgical operations [4]. The implementation of the notification code for reporting HIV and acquired immunodeficiency syndrome (AIDS) to protect patient privacy was adopted in 1994 [2]. Free voluntary counseling and test centers for HIV were established in Ankara, Istanbul, Izmir, and other provinces in 2005 [5]. In addition, the MoH published a comprehensive diagnosis and treatment guide in the same year. The corresponding legislative regulations have since then been continually published by the MoH and are mainly limited to prevention and protection measures. However, in other areas such as education, social service, and criminal liability, no specific regulations regarding HIV exist. In other words, HIV and AIDS-related issues are not mentioned in Turkish Law, except in MoH circulars [4]. As the recorded HIV incidence is relatively low in Turkey compared to other hard-hit countries, both policymakers and the community do not seem to consider it as an important health issue [6].

The financial and emotional burden that HIV lays upon a person differs from other transmissible and chronic illnesses [7]. Moreover, biases exist toward PLWH likely due to the fact that the illness was mostly observed in men who have sex with men (MSM), sex workers, and intravenous substance abusers in its early years of occurrence [8, 9]. PLWH may anticipate and experience stigma from different sources, such as friends and family, sexual partners, coworkers, strangers, healthcare professionals, and institutions. Healthcare professionals’ discriminative attitudes toward PLWH may cause distrust between patient and physician and, ultimately, unfavorable results such as poor adherence to the treatment process and discontinuation of medication [10]. Similarly, a growing body of literature suggests that being subjected to discrimination in both health institutions and social spheres imposes an emotional burden on the person, which might lead them to refuse treatment and put their physical and psychological health at risk [11, 12]. In addition, there have been cases where people have been fired from a job after being accused of having HIV infection or AIDS [13]. In studies from China and the Netherlands, employers were also found to be reluctant to hire people with HIV [14, 15].

PLWH have been exposed to stigmatization and discrimination since the HIV epidemic began, both in Turkey and worldwide. This, in turn, negatively affects PLWH’s access to healthcare services and impairs public health by causing inequality in terms of both the frequency and the quality of the services received [16]. Research has shown that in places with low HIV prevalence, there is a more intense strain on PLWH due to the fear of HIV [17]. In a study on the causes of HIV-related stigma, Koseoglu et al. (2020) showed that more than half of the treating physicians and nurses did not have any specific training on HIV-related issues, including stigmatizing attitudes toward patients. A majority of them believed that HIV can be transmitted by handshaking and/or via a droplet spread [18]. Similarly, in another study, healthcare professionals were found to be prejudiced regarding HIV-related issues and lack accurate, adequate knowledge about the disease, such as its contagiousness, mode of transmission, and sociodemographic risk factors [19]. Kose et al. (2012) showed that PLWH in Izmir, the third largest city in Turkey, experienced problems at work, in their social relationships, and regarding access to healthcare services [20]. Furthermore, in Turkey, HIV-related non-governmental organizations (NGOs) have periodically published reports of human rights violations. For example, according to a report published by the Positive Living Association (PLA) on the violations of rights among PLWH from 1985 to 2007, PLWH are subject to discrimination in health services, in family/social surroundings, and at work [21]. As per another report by the Pozitif-iz Association^1^ covering human rights violation incidents between 2018 and 2019, PLWH have limited access to healthcare and experience violations of their privacy in healthcare settings [23]. According to the latest report published by PLA, most commonly, the confidentiality of their personal information is compromised [24].

Campaigns directed by the Joint United Nations Programme on HIV / AIDS (UNAIDS) such as 90–90-90,^2^ Fast Track,^3^ and Undetectable = Untransmittable^4^ have led to significant progress in raising public awareness and prevention of the epidemic [28, 29]. However, in many countries, PLWH are still subjected to stigma and discrimination [30–32]. Although a similar trend in Turkey has been well documented [33–35], there are few studies on PLWH’s perception of their diagnosis and treatment processes, their relationship with healthcare professionals, and their experiences in healthcare environments. Therefore, this study focuses on PLWH’s perspectives regarding their illness experience while receiving healthcare services and explores how their sense of self is influenced after being labeled as “ill” as well as how their relationships with healthcare professionals, their immediate circles, their thoughts about their social status, and their lives in general are influenced during and after diagnosis.

## Methods

This study conducts a descriptive qualitative analysis of the lived experiences of PLWH in healthcare settings in Istanbul, Turkey. In in-depth interviews, a researcher (GS), asked participants to share their accounts regarding their experiences with physicians and other healthcare professionals; their families, friends, and colleagues; and healthcare institutions’ bureaucratic bodies and insurance companies in the process of being informed about their diagnosis and the planning of their treatment. Additionally, we inquired how their lives changed after the diagnosis and during the treatment. We examined their perceptions by coding the interviews thematically.

### Participant recruitment

Overall, we recruited and interviewed 20 participants in this study. An analysis of the interview logs showed that data saturation had already been achieved. In other words, since no new themes emerged, no further recruitment was necessary.

The research population were PLWH aged over 18 years and living with HIV for more than a year. We included the latter criterion, because we expected our participants to have gone through the time-consuming processes of testing, diagnosis, and beginning of treatment during which patients tend to face problems. We expected them to have reached a certain phase of the illness where they could view their experiences from a distance without having to deal with the initial difficulties, such as receiving the diagnosis and adapting oneself to the unexpected and/or shocking situation.

In this study, we used the snowball sampling method since we could not define an easily accessible sample, as the research necessitated reaching vulnerable people [36]. Therefore, initially, we contacted NGOs such as the Human Resource Development Foundation,^5^ the Pozitif-iz Association, and physicians specializing in clinical microbiology and infectious diseases who work on HIV to reach PLWH who might be willing to participate in the study. After recruiting these first-order participants, we reached other PLWH who met our criteria (the second order). We obtained informed consent obtained from all participants at the beginning of the study. We did not pay any financial compensation to them.

### Data collection

GS contacted the participants directly and performed face-to-face interviews in places where they were available or felt comfortable such as clinics, cafes, and participants’ homes. Only one of the participants preferred to be interviewed via Skype. One person refused to participate in the study because she/he distrusted the services she/he received at the institution with which the researcher was affiliated at that time. GS took field notes during and after the interviews about the surroundings, the participants’ moods, and unexpected interruptions. GS interviewed most participants individually except two marriage couples who claimed to be interviewed in pairs as they had been together during all the diagnosis and treatment processes. The interviews lasted 25 to 80 min, and 51 min on average. Repeat interviews were unnecessary.

We used an interview guideline form, which we developed through a literature review, for the interviews. Based on the results of three qualitative studies [20, 38, 39] we discussed interview questions with sociology specialists and modified them accordingly. We designed the in-depth interview guideline to ensure a logical flow and appropriate expression of questions and to minimize confusion among the participants. The key interview questions, which sought information about participants’ experiences, emotions, and actions, are presented in Table 1.

**Table 1:** Key questions

How did you find out you were positive after the tests were verified?	
How were you notified?	
How did you feel after the physician notified you?	
Was your partner with you at that time, and were they notified of the test results?	
Did you want to tell your immediate circle about the diagnosis, and what was their reaction?	
What reaction do you encounter when you go to a hospital for a health problem?	

### Data analysis

GS transcribed the voice recordings taken during the interviews verbatim into raw logs and assigned each participant a protocol number. Two researchers (GS and MVK) analyzed the raw logs thematically by using the inductive content analysis methodology. The approach is recommended, if former knowledge is inadequate or fragmented about a phenomenon. Accordingly, the explanatory or thematic categories are derived from the data. Inductive content analysis is represented in three basic phases: open coding, creating categories and abstraction [40]. The researchers carried out the coding manually without the use of software. The analysis includes seven steps, which are explained below.

First, GS conducted a rough reading of the raw logs to gain a general understanding of the interviews and to get familiarized with the overall data (Step 1). Next, she excerpted, sorted and clustered the expressions regarded as relevant answers to the key questions. In this step, she divided the excerpts into the smallest units of content integrity. Meanwhile, she took both the manifest content and the latent content into account (Step 2). Subsequently, GS and MVK worked on the data individually. They separately reviewed and grouped the responses under the suggested themes and created a draft thematic framework based on their own preconceptions and knowledge (Step 3). Next, the researchers gathered to discuss and structure the themes by crosschecking their findings to reach a consensus. For that purpose, they compared the suggested themes and reflected on their semantic similarities or differences in order to reconcile them in clusters (Step 4). Based on the revised theme-set, they created a themes, sub-themes, and codes table. At this step, they checked whether some concepts could be further clustered together according to their meaning-closeness and, if so, whether these clusters could be classified under the main themes (Step 5). Then, they defined the relationships between these elements and attempted to reconstruct the data on macro and micro scales. On a macro scale, they attributed a contextual framework (such as external factors affecting participants’ perceptions; e.g.: one’s relationships with her family members) to every distinct semantic group. On a micro scale, the researchers noted possible manifestations of those relevant experiences in the individuals’ lives (such as changes in their moods, attitudes or feelings) (Step 6). Finally, they interpreted findings in comparison to the data obtained from prior relevant studies to gain a comprehensive understanding (Step 7) [41].

## Results

Twenty people were interviewed (19 in person and one via Skype) between October 18, 2017, and April 29, 2018. The participants comprised 5 women and 15 men, from Istanbul, ranging from 27 to 55 years of age. Research participants had varied social status profiles and had been living with HIV for 1.5 to 20 years at the time of the study. The majority of them were single and associated with NGOs active in the field of HIV-related issues. The participants were not asked about sexual orientation. The number of male participants was significantly higher than that of female participants. However, the distribution of our participants by sex was in line with the data on the distribution of PLWH in Turkey by sex. According the report of the MoH, the rate of women and men living with HIV was found to be 20,1% and 79,9%, respectively (3). A summary of the demographics of the study participants is given in Table 2.

**Table 2:** Demographic information of the participants

Sex	Women (*n* = 5)	Men (*n* = 15)
Age	Average: 41,5	Average: 34,5
	Min: 34	Min: 27
	Max: 47	Max: 55
Occupation	Employed: 3	Employed: 13
	Unemployed: 2	Unemployed: 2
Education	Primary school: 1	Primary school: 2
	High school: 3	High school: 5
	University graduate: 1	University graduate: 7
		Doctorate: 1
Marital status	Single: 2	Single: 12
	Married: 3	Married: 3
Health insurance	General Health Insurance (GHI)^a^: 3	GHI: 11
	Private Health Insurance (PHI)^b^: 1	PHI: 4
	None: 1	
Time lived with HIV	Average: 10 years 2 months	Average: 5 years 7 months
	Min: 2 years	Min: 1,5 years
	Max: 20 years	Max: 14 years

^a^ GHI: This denotes the insurance that finances employees’ healthcare expenses and is used in state hospitals and health institutions. The unemployed may also have this insurance if they pay the insurance charges. It covers the diagnosis and treatment of HIV [2]

^b^ PHI: This is the customized insurance type determined according to certain limits and general requirements and used for healthcare expenses. It does not cover the diagnosis and treatment of HIV [42]

The type of health insurance that our participants benefited from varied. All the participants asserted that they do not use their private health insurance (PHI) for HIV treatment since it does not cover the treatment and/or insurance companies can notify their employers of their HIV status. One of the participants was a foreigner and did not have any health insurance.

We examined the results obtained from the thematic analysis in three contexts, which were attributed to the clusters of themes while mapping the thematic pattern (analysis step 6): (1) interactions with healthcare providers, (2) participants’ responses to their HIV diagnosis, (3) interactions with social networks.

### Interactions with healthcare providers

We classified physicians’ reluctance to inform the patient fully and their different approaches while doing so under this context. We found that the physician-patient relationship to be a determinant factor in patients’ perception of their illness process. Furthermore, the experiences of the participants suggested that they were exposed to confidentiality and discrimination in healthcare services.

#### Withholding information from the patient

The majority of the participants stated that they were tested for HIV without their knowledge when they went to the hospital for other health problems or to get an official health report required from every couple before marriage. Moreover, they asserted that they were not informed about the preliminary test results when they were asked to give a blood sample for verification; thus, they felt that healthcare professionals withheld information from them.

“We need to redraw blood from you.” And I asked why? They said they couldn’t tell it at that moment. It was exactly like that. I said “You have to tell me because you requested me to come again. You want to draw blood, it’s my right to know.” “No, we don’t want to turn your stomach by saying it now.” That’s exactly what they said. (3 years since HIV diagnosis)

#### Informing the patient about diagnosis

We deemed notifying the patient on the phone, informing the patient immediately after diagnosis about the potential HIV-related problems, and not giving complete information or giving wrong information as inappropriate approaches.

[The doctor] told me that I should hide this from my family, I shouldn’t be so enthusiastic about finishing school, and because of this, my work life could be affected. He even advised me to suspend school for some time until I pulled myself together. I was shocked but still had the impression that what he said was not so logical but horrifying. (6 years since HIV diagnosis)

Moreover, we categorized informing the patient in the presence of family members and not being attentive in that process as inattentive approaches.

Two young doctors came to my room and told me the diagnosis when my mother was there. Now, I think that it’s a very serious violation of rights. I may or may not want to share it with my mother. I don’t think adequate attention is paid to the patient’s confidentiality. (3.5 years since HIV diagnosis)

In contrast, we evaluated physicians preferring an isolated environment to notify the patient and to inform them about the test results as an attentive approach.

( … ) [the doctor] was so friendly and answered my questions one by one by building such a healthy relationship and informed me in such an appropriate manner that I realized guidance like this relieved me a lot. I mean, I saw how precious it is to have a good relationship with the doctor and to get counseling services during the diagnosis. (3.5 years since HIV diagnosis)

#### Physicians’ attitudes toward the patient

Participants stated that the physicians’ positive or negative attitudes toward them influenced their treatment processes and led to changes in their lives after the diagnosis. They deemed the physician misinforming them about the treatment process and medication use, estimating their survival time, and not establishing open communication with them as indicative of negative attitudes.

[The doctor] said he could keep me alive for another 15 years. “What do you mean?” I asked. I was 22 then; add 15, it makes 37. I imagined not being able to see my 40th birthday. I felt so bad. I thought of my mother. I left the room immediately. I was baffled and didn’t know what to do. I felt very upset. (7 years since HIV diagnosis)

In addition, the participants also stated that certain behaviors on the part of physicians caused them to feel humiliated.

“You sit down there,” she said to me. She opened the windows and the door, moved away from the desk, and took a piece of paper. It was only 15 minutes since I got the diagnosis. “How old are you? Are you homosexual?” she asked me directly. Then, she asked if I drank alcohol, smoked, had a nightlife, had had a lot of partners in the past six months … Well, at first, I had a humming noise in my head, but then it faded, and it turned to curiosity; I wondered what this woman’s intention was. ( … ) I remember her reopening the window when it got closed a couple of times. ( … ) Then, I left the room and tore the test results into pieces. (12 year since HIV diagnosis)

Several participants stated that instead of making a mutual decision, they followed the physicians’ orders to avoid any problem from occurring. One of them, for example, said he could not object to his physician’s request to take his photo just because he was a physician.

He asked if he could take my photo, and I asked why. “As part of the follow-up; to keep record of your initial state, the treatment process, and afterward,” he said. I asked if I was in a bad state at that time and why he put it as now, before and after. He told me that he wanted to follow up on his patients visually. ( … ) He took a photo of me, which made me crazy, but I thought I shouldn’t oppose; after all, he was a doctor. (3 years since HIV diagnosis)

Most of the participants asserted that physicians and other healthcare professionals working at infectious disease clinics treated them sympathetically, provided professional guidance to them regarding their future life, and referred them to NGOs active in the field.

“Look,” he said, “we are here with you whenever you have a medical problem.” He handed me a piece of paper in which there was information about the foundation. He said, “You can contact them; they have a good counseling service. You can find any information you need there.” Then I received relevant information from them. (3 years since HIV diagnosis)

#### Failure to protect confidentiality

The confidentiality of all the participants was not fully protected in healthcare environments. One reason for this is the failure of the healthcare system, health databases, and PHI companies to take the necessary precautions to protect the confidentiality of the personal data/information of people receiving services from them. Our participants’ accounts corroborated this information.

I have private health insurance as well, but I didn’t use it for this illness because they immediately notify the institution where I work [about my HIV status], so I use my general health insurance for this. (3 years since HIV diagnosis)

In addition, participants stated that healthcare professionals did not pay enough attention to protecting patient confidentiality and they frequently faced this problem, especially in physicians’ clinics and pharmacies.

There were two other customers waiting next to me. I was sitting on a chair when the pharmacist asked the pharmaceutical technician to bring three boxes of X by shouting out the names of the drugs. Then, speaking loudly again, he said “Your medication is ready, Mr. Y.” The one sitting beside me stood up, and I understood at that moment that he was living with HIV. (3.5 years since HIV diagnosis)

Moreover, the participants expressed that some health professionals ask questions such as “how did you get the virus?” out of curiosity, and they felt this reflected an unprofessional attitude.

I went to see a psychologist for the first time. Anyway, I was talking to her, telling her about it. She asked me how I got the virus. People are very curious about it. What’s it to you! (2 years since HIV diagnosis)

#### Being subjected to discrimination

The main concerns of the participants in this regard are healthcare professionals’ reluctance to operate on PLWH, taking excessive precautions before any medical intervention, scaring the patients, not seeing them as people, and feeling pity for them.

I went there for a dermoid cyst problem. ( … ) but I said I was HIV positive. He said, “I don’t operate on HIV positives.” “Why not?” I asked him, “Do I have a different chromosome structure?” He said they wouldn’t be able to provide the necessary environment to prevent infections. I didn’t get it. I sensed that he was abstaining. But I didn’t buy it—tell another lie! (6 years since HIV diagnosis)

In addition, most participants expressed that relevant regulations should be developed so that PLWH could receive healthcare services from health institutions just like other patients without being subjected to any discriminatory acts and that healthcare professionals should be more attentive to behaving in a manner showing an understanding of their condition.

As I said before, I went to a hospital, a private one, after I came back. There, a doctor said, “Don’t tell anyone about your illness, don’t go to state hospitals, and don’t give blood sample because they would fire you once they found out.” I felt horrified. Where should I have gone? ( … ), then I didn’t give a blood sample no matter who requested it. I lived like this for three years. (8 years since HIV diagnosis)

Some of the participants also stated that they think healthcare institutions do not want to provide service to them and that healthcare professionals working at private healthcare institutions are not sufficiently knowledgeable about HIV.

I had an epidermal cyst, and in order to have it removed, I went to the surgery clinic of the hospital where I get HIV treatment. They told me that they were too busy and referred me to another hospital. I went there ( … ) They said that providing healthcare services to such patients is not welcomed at their hospital. They told me “Go the hospital where you get treatment for it and have yourself operated on there.” (11 years since HIV diagnosis).

### Participants’ responses to their HIV diagnosis

Participants’ experiences related to blaming oneself after the diagnosis, experiencing hardship in confronting the disease, and feeling the fear of not being taken care of due to being diagnosed as HIV+ were the main themes we examined in this context. Their concerns centered on whether to share their diagnosis with others, adherence to treatment, decisions regarding sexual activity, and issues surrounding parenthood. The participants used a variety of coping strategies for the problems they faced in their diagnosis and treatment processes.

#### Participants’ feelings

The participants’ main source of information about HIV was the news on television and in the newspapers. Some of the participants stated that they had never thought they would have AIDS since, according to their view, it is a disease that homosexuals and sex workers have.

( … ) over the years so many pieces of information accumulate in your head, in your mind. Out of the blue, I pictured a scene: a famous journalist is bargaining with a blonde woman sex worker, she says she has AIDS. ( … ) I told myself that I was neither blonde nor a sex worker; this couldn’t be possible; such a thing wouldn’t happen to me. (14 years since HIV diagnosis)

However, the participants who did not share this opinion and those who had proper knowledge of HIV tended to blame themselves as they failed to protect themselves.

You experience a huge intrinsic stigma. “How can it be possible when I have so much knowledge about it?” You keep asking yourself. You get mad at yourself and blame yourself. (3.5 years since HIV diagnosis)

The participants did not want to acknowledge their condition for a long time after the diagnosis; some even refrained from seeking treatment. Moreover, due to the discriminating instances in healthcare institutions they were aware of, some participants developed a fear of being rejected by hospitals when they needed to undergo an operation.

I don’t know what to do if I need to undergo an urgent operation. I have concerns. ( … ) Since I’m also a physician, I’m aware of some things. I know there are persons who have not been operated on. This could happen to me, too. (2 years since HIV diagnosis)

#### Changes occurring in the patient’s life after diagnosis

One important change in participants’ lives after their diagnosis was ceasing their sexual activity.

( … ) I haven’t had any [sexual intercourse] for 14 years. I don’t want it. Anyway, I can’t find anyone like me. It’s difficult, especially in this country. (15 years since HIV diagnosis)

In addition, participants tended to isolate themselves from social interactions considering the possibility of being exposed to discrimination and fearing transmitting the virus to other people because they did not know the modes of its transmission.

“Why bother? I’ll die in any case, so I should die without disgracing myself,” I said to myself. If the doctor also has this projection [that I will die], if the one who is supposed to support me treats me badly, how can I talk about it publicly? I don’t have to explain it, though. If my life is bound to end, it will. Then, I didn’t leave home for several days. I thought I could change the city I lived in; then no one would know me. And I left the city. (3 years since HIV diagnosis)

#### Patients protecting themselves against adverse consequences of the disease

The participants tended to hide their HIV status in healthcare settings and their healthcare experiences to avoid negative reactions from both healthcare professionals and other people.

Of course, when I go to a dentist, I don’t tell him, “For your information, I have this.” So, I don’t experience such things. I don’t explain that I underwent this when I go to an ear, nose, and throat clinic for a cold or flu. I don’t have to! I just explain the complaint for which I go there, and they do what they need to do. (1.5 years since HIV diagnosis)

Some of the participants stated that they attempted to find a new healthcare professional when they faced a problem, strived to stand up for their rights.

( … ) only after some struggle, after going to the chief physician and telling him that I had been rejected, and with his intervention, was I able to have the surgery performed. (11 years since HIV diagnosis)

Some participants received help from foundations providing support to PLWH.

One evening, I had a problem with my medication. There was no one at the hospital that we could reach at that moment. It was a state hospital; whom could I call? I wasn’t getting treatment from a private hospital, so I didn’t have any contacts there either. What do we do in such cases? We call the foundation. (3 years since HIV diagnosis)

### Interactions with social networks

In this context, changes occurring in the participants’ lives and in their relationship with their family members and friends were the main themes. According to the participants, problems regarding how PLWH’s souses/partners are notified of their diagnoses emerged as a prominent issue. This is done in a variety of ways, some of which could be quite controversial.

#### Changes in family dynamics

The participants stated that their family relationships were affected either positively or negatively after they shared their HIV status with family members. Some participants expressed that their families were supportive.

How well they looked after me and how much attention they paid to me... ( … ) I thought I had to get better. I almost went crazy seeing them feeling sorry for me. It’s the same now; they call me every minute. They tell me not to worry no matter what happens. Thanks to them… (6 years since HIV diagnosis)

Four of the participants stated that their families ended their relationship with them and put the blame for contracting the disease on the lifestyles they pursued.

I came back to Turkey so that my family wouldn’t find out. They found out later, of course. They told me “You’ve been changing lovers all the time; you’ve been on the loose; you deserved it.” I’ll never forget that. (20 years since HIV diagnosis)

Some participants, however, decided not to share their diagnosis with their families.

I didn’t want to share it. Well, it was because they don’t have awareness of this disease and haven’t encountered it before, and they aren’t healthcare workers. I mean, everything is under control; what needs to be done is being done at the moment. I didn’t want to upset them. (2 years since HIV diagnosis)

#### Changes in friendship dynamics

The participants’ decision to disclose their HIV status was influenced by social dynamics. Although most of the participants said they were supported by their friends after diagnosis, several stated that their friendships ended and their friends avoided close contact with them.

I lost only one of my friends. He was a really close friend that I really liked. He said, “Dear, I’m so sorry too, see you later.” But he never called again. How long? It’s been about ten years since we last met. There wasn’t any problem between us. ( … ) He should have called to see if I died or not. I got really upset, but, of course, there was nothing to do. (12 years since HIV diagnosis)

The majority of the participants asserted that they had not had any problems with their friends, and their illness had not affected their relationships.

When I was in the hospital, I had lots of visitors and phone calls. It seems that I had lots of people who loved me. I always had flowers in my room, lots of calls and messages on my phone. (14 years since HIV diagnosis)

#### Spouse/partner notification after HIV diagnosis

As stated by the participants, various methods were used for spouse/partner notification. They favored some of these methods, while they thought that others negatively influenced their relationship with their spouse/partner. For example, they were pleased when physicians encouraged and supported them to notify their spouse/partner. They also appreciated when physicians informed their spouse/partner together with them.

I said [to the doctor] that I wanted to break up with my fiancée since I couldn’t tell her I had such a disease. He said, “just bring her over, let’s talk, then maybe she accepts.” I did so, and he talked to her. If he hadn’t talked to her, perhaps we would not have been able to get married. Now, my wife is the only one that I lean on. (3 years since HIV diagnosis)

One of the problems expressed by our participants was the inability to notify their partners. For example, single participants stated that they did not know exactly when they had been infected and, thus, could not contact all of their ex-partners. Those with multiple partners said they did not know the people they had had sexual intercourse with very well, so it was difficult to contact them. Further, due to an “infidelity” aspect in the case of married participants, they feared the possible breakup of their marriage/relationship, which deterred them from notifying their spouse/partner.

Well, I had sex with another woman. I got this from her. Had I known, would I have done it? I didn’t tell my wife. I have two daughters. I didn’t tell her so as not to break up my family. After many years, she found out, but she wasn’t infected. After all, she divorced me anyway. (5 years since HIV diagnosis)

Some participants stated that their spouse/partner was notified by their physician without their knowledge, which damaged their relationship.

No, I didn’t know. ( … ) I mean I wasn’t hiding it, but I should have told her myself. [The doctor] told it to my girlfriend. ( … ) I’d rather have told her myself, instead of the doctor, and then we talked what was what anyway. ( … ) She got scared. We had a small crisis, but we got over it. (7 years since HIV diagnosis)

Moreover, one participant told his wife that he had another illness to hide the actual diagnosis.

I went abroad for business and stayed there for three years. They told me I contracted HIV there. Then, I came back here. ( … ) To be honest, we [I and my doctor] told my wife that I had hepatitis. We had her and the kids tested for HIV as if they were at risk of developing hepatitis. Thank God, the test results were negative. And that’s it. But my prick conscience still aches. (3 years since HIV diagnosis)

## Discussion

In the present study, we analyzed PLWH’s perceptions of their relationships with healthcare professionals, family members, and friends as well as their accounts of the changes in their lives based on their lived experiences at the onset of their illness. In light of our findings, we centered the discussion on the following themes: autonomy and obtaining informed consent, the right to privacy and private life, discrimination and stigmatization in the healthcare setting, the impact of HIV on personal and social life, responsibility for third person and spouse/partner notification.

The majority of our participants found that they had HIV when they went to the hospital for other health problems or to get the medical reports required before marriage. Our findings suggest that the participants experienced a shock at this stage because the test was performed without their knowledge and the results were delivered to them without any accompanying counseling services. In both Turkey and other countries there seem to be several barriers against HIV testing and diagnosis. Due to the lack of general awareness, knowledge in the society, the stigmatization of PLWH, the shortage of HIV testing and counseling centers, patients are likely to be left without support as they face problems such as societal discrimination, anxiety, and marginalization [43–46].

According to the results of a survey carried out by Gokengin et al. (2017) on PLWH in the cities of Istanbul, Ankara, and Antalya, 52% of the participants got tested for HIV without their prior knowledge and consent. Further, 77% of the participants were not offered any counseling services during the testing process while 21% received it after diagnosis and 2% were offered such a service both before and after diagnosis [38]. In another study, the authors conclude that individuals taking voluntary HIV tests is crucially important for both the patient and the community [47]. However, there are obstacles to fulfilling this objective. The shortcomings in the healthcare system in Turkey, such as the restricted time allocated to patients, heavy workload of physicians, lack of professional experience in disclosing the diagnosis to the patient, and lack of competence in communication and interpersonal skills may create certain problems in informing and notifying the patient [42, 48]. Research suggests that the quality of the physician-patient relationship is a significant factor influencing a patient’s desire to pursue treatment [49–51]. The participants highlighted the significant effect of the physicians’ attitudes on their lives and treatment processes. Their statements suggest that most of them had a good relationship with their physicians. Some participants, however, stated that their doctors did not inform them properly about their diagnosis and treatment plans; moreover, they exhibited avoidant behaviors. Since such difficult conversations are a form of disclosing the diagnosis, the physician should take adequate time to talk to the patient and should do so in person unless there is an obstacle precluding this option. Second, for the same reason, the physician should not give all of the information to the patient at once but, rather, wait for her to digest the bad news before continuing with further details [52]. An essential component of a healthy physician-patient relationship is the physician’s ability to provide appropriate professional guidance to their patients. PLWH suffer from being subjected to intersectional stigma, “a concept that has emerged to characterize the convergence of multiple stigmatized identities within a person or group, and to address their joint effects on health and wellbeing” [53], p:1). In the case of PLWH, physicians are expected to be aware of this phenomenon and approach patients accordingly. For improved communication between the physician and the patient, it is imperative that undergraduate and graduate medical education in Turkey covers subjects such as a professional approach to vulnerable groups and communication skills to ensure that the physicians confront their preconceptions of PLWH [53]. This is important not only to facilitate physicians’ awareness of their attitudes contributing to or reinforcing the intersectional stigma but also to facilitate their ability to help their patients cope with being diagnosed as PLWH and the illness in a supportive and protective manner.

The majority of the participants stated that healthcare professionals notified their employers, families, and friends of their HIV status without their prior knowledge or consent. Similar results were reported in a survey conducted by Gokengin et al. (2017). While 43.9% of the participants said they were sure that their HIV status had been disclosed to another person and institutions by healthcare providers without their consent, another 30.6% of them had suspicions in this regard [38]. Not protecting the confidentiality of personal data is frequently observed in other counties as well, especially in the case of PLWH [54–57]. Informing the patient’s family about the diagnosis without the patient’s consent is against the principles of respect for patients’ autonomy and protection of their privacy. However, there is no legislation regulating how family members should be informed in such cases [58].

In addition, some participants stated that their health insurance companies shared their HIV test results with their employers. Although it is not a legal obligation, most employers in Turkey require HIV testing from their applicants as a pre-requisite for recruitment [13, 59]. It is known that PLWH feel threatened at work, are harassed in the workplace, and even get fired after diagnosis. In addition, practices such as not employing such people, and forcing them to resign burden them with economic problems and social dilemmas. Moreover, there are cases where PLWH quit their jobs due to negative social reactions, stigmatization, discrimination, and guilt [60]. In a study investigating workplace problems that PLWH face, it was found that once PLWH lost their jobs, they were largely unable to find new jobs due to their HIV status [13]. According to another study, the employability of PLWH was significantly linked to age, time since diagnosis, illegal drug use record, and cluster of differentiation 4 (CD4) counts. Younger PLWH are more likely to be involved in the workforce. PLWH’s employability was not substantially linked to education level [61]. Most counties have various legislative regulations to prevent the discrimination of PLWH in their workplaces [62, 63]. According to the code of practice specified by the International Labour Organization, there should be no discrimination against PLWH in the workplace, and employees with HIV should be able to work as long as they are medically fit [64].

Outside of healthcare institutions, the use of personal health data by employers, payment providers, and health insurance companies is acceptable as long as they serve a useful purpose such as meeting the needs of the patients, improving the healthcare system, and protecting public health. However, for sensitive groups, it brings about problems in terms of the protection of confidentiality and arouses questions as to with whom and to what degree such data should be shared [65]. As of 2003, with the implementation of the Health Transformation Program, the private sector began to play a bigger role in the delivery of healthcare services [42], and personal health data have been collected by the MoH, the Social Security Institution, and PHI companies. As a result, the rate of healthcare services provided by private companies has increased, which poses the risk of usage of health data for commercial purposes. This makes personal health data accessible to a third person and paves the way for their disclosure at the national and international levels [66]. Similar examples have been reported in literature [67–69]. Patients should be informed of what information is recorded and what protection is available to prevent the disclosure of this information. Some of our participants stated that their physicians referred them to the foundations where they could receive consultancy for such problems. However, NGOs may not be able to reach all such people at the same time. Therefore, such services should be offered as part of public services at hospitals and other healthcare institutions.

HIV-related stigma within the healthcare setting is known to influence the utilization of avoidance and treatment administrations [70]. The participants stated that they faced rejection or were met with excessive precautions when they went to a hospital for another health problem. They deemed such behaviors a form of discrimination. Likewise, it has been reported in studies conducted in different countries that the participant PLWH were subjected to discrimination in healthcare environments and thus refused to seek healthcare services [71–74]. There are also studies in Turkey that demonstrated the stigma and discrimination in healthcare settings at both institutional and individual levels against PLWH [34, 38]. The report issued by the PLA regarding the violation of rights revealed that PLWH are subjected to discrimination primarily in healthcare environments, they are not given the necessary medical care, and their right to health is impeded [75]. In a study which examined healthcare professionals’ attitudes, it was found that preconceptions and a lack of knowledge of the modes of transmission and prevention were high among all participants, and 50% of the participants stated that they did not want to follow up with PLWH [19].

Living with HIV may pose a significant risk factor for a person’s private life and psychological health [76]. Since the disease is prevalent among individuals subjected to discrimination in society, such as sex workers, and drug users, as per interviews with the participants that this may have led to biases and anxieties in them regarding HIV. In this context, the process of notifying patients of the test results is particularly highlighted. Being diagnosed with HIV may lead to confusion, anger, sorrow, and fear in individuals, even if they already suspected it [77]. Similarly, in our study, the participants experienced a sudden emotional trauma upon receiving the diagnosis and thus could not rationally evaluate what the physicians were telling them. For instance, the belief that HIV is a disease of homosexuals and sex workers caused most of our participants to blame themselves and develop a fear of rejection and stigmatization, making them reluctant to acknowledge their diagnosis. Struggling with the lifestyle changes caused by the disease, such as ceasing all sexual activity, avoiding close contact with people, and not being able to talk about their HIV status with their families or friends on their own, may put serious pressure on the patients. According to the findings of a mixed method study carried out in Turkey in 2002, the biggest challenges PLWH faced were being subjected to discrimination, difficulty in sharing their HIV status with others, impaired sexuality, and financial issues. In the face of such problems, they mostly used palliative coping strategies and developed anger [39]. According to another in-depth interview study conducted with 27 PLWH in İzmir, Turkey, in 2018, the patients tended to keep their HIV status to themselves due to the discriminative and stigmatizing incidents in society [78]. Our findings are in line with the results of these two studies.

It is known that PLWH are mostly willing to share their HIV status with their partners and their family members to gain emotional support from them, as well as taking precautions against transmission [39, 79]. Considering that PLWH should be able to decide when and how their close circle should be informed of their diagnosis and that they might actually need the support provided by their family members and friends, the experiences of PLWH and healthcare professionals regarding conversation about the diagnosis should be further studied. A deeper understanding of the conditions and needs of both parties might inform efforts to produce guidelines and/or regulations on notifying patients’ social spheres.

Since HIV is a sexually transmitted virus, problems arise between the patients and their spouses/partners. It is ethically problematic that the PLWH desire not to disclose their personal data and thus keep their spouses/partners or persons they have sexual interaction with uninformed about the risk that they pose to them [80–82]. Although partner notification (PN) can save the lives of people facing the risk of being infected with HIV, it constitutes certain obstacles for the physicians and their patients that hinder its practice. For example, the lack of any legal framework on this issue might compel physicians to make personal decisions by acting at their own discretion [4]. In public hospitals in Turkey, PLWH are given an informed consent form for PN. First, they are verbally informed that they need to notify their partners. If the PLWH do not inform the physician that their partners have taken an HIV test, they are given a written warning reminding them of the need to notify their partners and stating that they can be provided with psychiatric support in this process and that their partners will receive a medical notification if they do not respond to the form [58]. The legal regulations applied to this issue vary across regions in countries. Some countries have legal obligations that enforce PN, others have regulations making it an optional procedure, and some do not have any regulations [83, 84]. In Turkey, people are prosecuted under general legal provisions in such cases [85]. However, rather than trying to prevent HIV transmission by prosecuting PLWH for transmission of the virus, governments should prioritize developing programs that have been proven to be effective in reducing HIV transmission and allocating resources for this matter. In this way, not only would the dilemmas that PLWH face would be solved but the HIV-negatives’ health would also be protected.

In light of these findings and considering the increasing number of HIV cases in Turkey, we think that it is crucial to develop curricula for medical and other health faculties to ensure that they take a professional approach toward PLWH to prevent discrimination against them in healthcare environments. In particular, HIV-related stigma and discrimination in the healthcare setting cause challenges for PLWH in regard to their access to healthcare services and thus pose a risk to their health. To prevent possible miscellaneous harm from occurring, the current regulations on patient rights must be put into effect. As deduced from our findings, people benefiting from counseling services tend to take action to pursue their rights when faced with a problem. In response to their pursuit, campaigns to raise PLWH’s and their families’ awareness must be conducted and centers that provide country-wide professional counseling services should be established.

### Research limitations

One of the difficulties we faced in this study was in reaching PLWH. This stemmed from their wish to not disclose or share their HIV status. Therefore, we paid utmost attention to ensuring that the participants did not feel nervous or prejudice directed at them. Overall, we do not think that they experienced such feelings because the participants who were not related to an NGO conveyed that they felt relieved talking about such issues with the interviewer, as they usually hesitated to do so with others. Furthermore, some participants whom we had contacted via an NGO expressed that the interviewer could call them later if any more information was needed. The interviewer also asked every participant whether they were disturbed by any of the questions or behaviors immediately after the interviews.

The participants who were associated with an NGO seemed more aware of their rights and were more eager to protect them than those who were not. Further, they were more at ease with receiving support and consultancy from the NGOs with which they were associated. However, many participants in the latter group independently told the interviewer that they had previously shared their situation with very few people. In addition, the experiences of PLWH in Istanbul and of those in other Turkish cities may differ significantly. Istanbul is known to be a modern cosmopolitan city where most people can express their identities explicitly, whereas in many other relatively small cities, this may not be the case. PLWH may encounter more severe stigmatizing and discriminative attitudes, which may originate from the predominant conservatism in smaller provinces.

Another limitation of the study was that the data obtained from the two married couples who participated in our study might have been affected since they wanted to be interviewed together. It was observed that male participants could not express their feelings openly in the presence of their spouses. These two couples were together in pairs during all diagnosis and treatment processes, and they reminded each other of incidents they thought would be good to mention during the interview.

We considered that people might be hesitant to talk about sexual preferences in Turkey. We chose not to ask questions related to this issue so that the communication between the interviewer and the participant was not hampered and was carried out smoothly. This may have affected the data obtained to some extent.

## Conclusions and suggestions

Our results suggest that the magnitude of HIV-related problems and their prevalence in Turkey remain tentative. The poignancy of the obtained results emphasizes the need for further studies representing the entire global population to be carried out on a larger scale.

Considering the perceptions of PLWH regarding their lived experiences during and after HIV diagnosis, we suggest developing and implementing informative and/or educational campaigns to raise public awareness, foster public engagement, and increase people’s knowledge of the issue, even though these endeavors might not be sufficient to reduce the HIV-related stigma in most settings globally*.* In addition, to encourage individuals to be tested voluntarily, pre- and post-testing counseling services designed in line with international guidelines should be easily accessible for individuals, and the number and quality of such services in hospitals and other healthcare institutions should be increased. It is essential that precautions are taken to protect PLWH’s personal health data against improper accessibility by third parties. The development and enforcement of a legal regulatory framework for PN would ease the burden of responsibility that both physicians and patients experience. In the fight against discriminatory and stigmatizing attitudes in healthcare environments—a problem frequently expressed by our participants—all healthcare professionals should be given professional training, and in case of any negligence, the necessary efforts to make up for such acts should be made per the legal regulations and procedures. Increasing the awareness regarding making the institutions providing clinical ethics support for the ethical conflicts that healthcare professionals and patients’ families experience may contribute to the protection of the professional values of physicians and ensure the benefit of the patient.

In conclusion, combating the stigmatization of PLWH requires multidimensional, multifarious efforts in a society. These include the improvement of patient–physician interactions, respecting patient confidentiality and privacy, improving healthcare provision by institutionalizing partner notification in order to balance patient autonomy, increasing public awareness, and providing public engagement to produce a purposeful legal framework based on ethical values.

## Data Availability

The qualitative datasets generated and analyzed during this study are not publicly available to avoid the risk of identifying individual participants.

## Abbreviations

AIDS: Acquired Immunodeficiency Syndrome
CD4: Cluster of Differentiation 4
GHI: General Health Insurance
HIV: Human Immunodeficiency Virus
MoH: Ministry of Health
MSM: Men who Sex with Men
NGOs: Non-Governmental Organizations
PHI: Private Health Insurance
PLA: Positive Living Association
PLWH: People Living With HIV

## Footnotes

1 The Pozitif-iz Association is a non-governmental organization that aims to strengthen PLWH and their families in physical, psychological, and social regards [22].

2 In 2016, the United Nations General Assembly’s Political Declaration on Ending AIDS submitted nations to the 90–90–90 targets, which planned to bring HIV testing and treatment to most individuals living with HIV before the end of 2020 [25].

3 The Fast-Track approach implies utilizing advancement to grow administrations, to more readily address individuals’ necessities and viewpoints, and to shine a spotlight on the areas and populaces with the most elevated HIV trouble [26].

4 Undetectable = Untransmittable (U=U) is a movement that demonstrates how to stop the sexual transmission of HIV [27].

5 The Human Resource Development Foundation is a non-profit, non-governmental organization that attempts to resolve the health, education, and employment problems that have a negative effect on the economic, social, and cultural development of human resource [37].
